# Automated Identification of Core Regulatory Genes in Human Gene Regulatory Networks

**DOI:** 10.1371/journal.pcbi.1004504

**Published:** 2015-09-22

**Authors:** Vipin Narang, Muhamad Azfar Ramli, Amit Singhal, Pavanish Kumar, Gennaro de Libero, Michael Poidinger, Christopher Monterola

**Affiliations:** 1 Singapore Immunology Network, Singapore; 2 Institute of High Performance Computing, Singapore; Johns Hopkins University School of Medicine, UNITED STATES

## Abstract

Human gene regulatory networks (GRN) can be difficult to interpret due to a tangle of edges interconnecting thousands of genes. We constructed a general human GRN from extensive transcription factor and microRNA target data obtained from public databases. In a subnetwork of this GRN that is active during estrogen stimulation of MCF-7 breast cancer cells, we benchmarked automated algorithms for identifying core regulatory genes (transcription factors and microRNAs). Among these algorithms, we identified K-core decomposition, pagerank and betweenness centrality algorithms as the most effective for discovering core regulatory genes in the network evaluated based on previously known roles of these genes in MCF-7 biology as well as in their ability to explain the up or down expression status of up to 70% of the remaining genes. Finally, we validated the use of K-core algorithm for organizing the GRN in an easier to interpret layered hierarchy where more influential regulatory genes percolate towards the inner layers. The integrated human gene and miRNA network and software used in this study are provided as supplementary materials (S1 Data) accompanying this manuscript.

## Author Summary

A gene regulatory network (GRN) represents how some genes encoding regulatory molecules such as transcription factors or microRNAs regulate the expression of other genes. Researchers commonly study GRNs involved in a specific biological process with the aim of identifying a few important regulatory genes. In higher organisms such as humans, a regulatory gene regulates multiple target genes and correspondingly any gene is regulated by multiple regulatory genes. Due to such multiplicity of interactions, a GRN usually resembles a tangled hairball wherein it is difficult to identify few most influential regulatory genes. In this study, we show that network analysis algorithms such as K-core, pagerank and betweenness centrality are useful for identifying a few important or core regulatory genes in a GRN, and the K-core algorithm is also useful for organizing regulatory genes in a hierarchical layered structure where the most influential genes in a GRN are found within the innermost layer or core. These few core regulatory genes determine to a large extent the expression status of the remaining genes in the network. We illustrate a pragmatic application of this technique to GRNs reconstructed from genome-wide gene expression measurements in the MCF-7 human breast cancer cell line.

## Introduction

Gene regulatory networks (GRN) are model representations of how genes regulate the expression levels of each other. In transcriptional regulation, proteins called transcription factors (TFs) regulate the transcription of their target genes to produce messenger RNA (mRNA), whereas in post-transcriptional regulation microRNAs (miRNAs) cause degradation and repression of target mRNAs. These interactions are represented in a GRN by adding edges linking TF or miRNA genes to their target mRNAs. Since these physical interactions are fixed, we can represent a GRN as a static network even though regulatory interactions occur dynamically in space and time.

A GRN provides a systemic view of gene regulation by coordinated activity of multiple TFs and miRNAs and thus serves as a medium for understanding the mechanism of gene regulation. In a biological process specific genes are switched on (activated) or off (repressed). Analysis of GRN can help in identifying important or core regulatory genes (TFs and miRNAs) that play significant role in controlling the specificity of gene expression during a biological process [1,2]. These core regulatory genes are candidates for further experimental investigation and potential targets for therapeutic intervention [3–5]. Analysis of GRNs also enables quantitative modeling of gene expression which can be used for rational design of molecular approaches to target specific biological processes [6] and infer new biology [7,8].

While the analysis of GRNs is well described in bacteria and yeast [9,10], similar analysis in higher organisms such as humans is challenging for a variety of reasons. Firstly, our knowledge of regulatory interactions between genes is incomplete, which is further complicated by the fact that the interactions may vary across different tissues [11]. Secondly, GRNs in higher organisms are highly complex as each regulatory molecule has dozens to thousands of targets and correspondingly a gene is usually targeted by multiple regulators. There is also cross-regulation and auto-regulation among genes. Such multiplicity of interconnections and loops makes the human GRN resemble a tangled hairball which is more challenging to analyze than a yeast gene network [12,13]. Lastly, gene expression is regulated at multiple levels in higher organisms and thereby transcriptional and post-transcriptional regulations represent only a fraction of total regulatory apparatus [14]. Hence gene expression cannot in principle be entirely explained using static GRNs.

As a result, although many studies construct GRNs in higher organisms, especially in human, methodologies for downstream analysis of GRNs are not well established [15]. For instance, a recent review reported that network analysis based methods for prioritizing candidate genes for disease gene discovery are still in infancy in contrast to gene set analysis based methods such as Gene Ontology and GSEA [16]. Research involving human GRNs has used a variety of approaches for qualitative analysis such as identifying important hubs, network motifs, hierarchical organization, pathways, etc. [17–20]. While this is useful for gaining intuitive insights into the functioning of a GRN, it is important to have robust and well characterized automated and quantitative analysis methods for gleaning useful information from human GRNs [21,22].

In view of the above challenges, this study aims to describe automated algorithms for analyzing human GRNs and organizing them into a meaningful structured hierarchy which is easier to analyze and interpret. We describe (1) the construction and analysis of an integrated network of human genes and miRNAs, (2) benchmarking algorithms for identifying core regulatory genes, and (3) algorithms for hierarchical organization of GRNs. We also discuss some practical considerations in the analysis of high throughput gene expression datasets in the context of GRNs.

## Results

### Construction of an integrated general human gene and miRNA network

**Fig 1:**
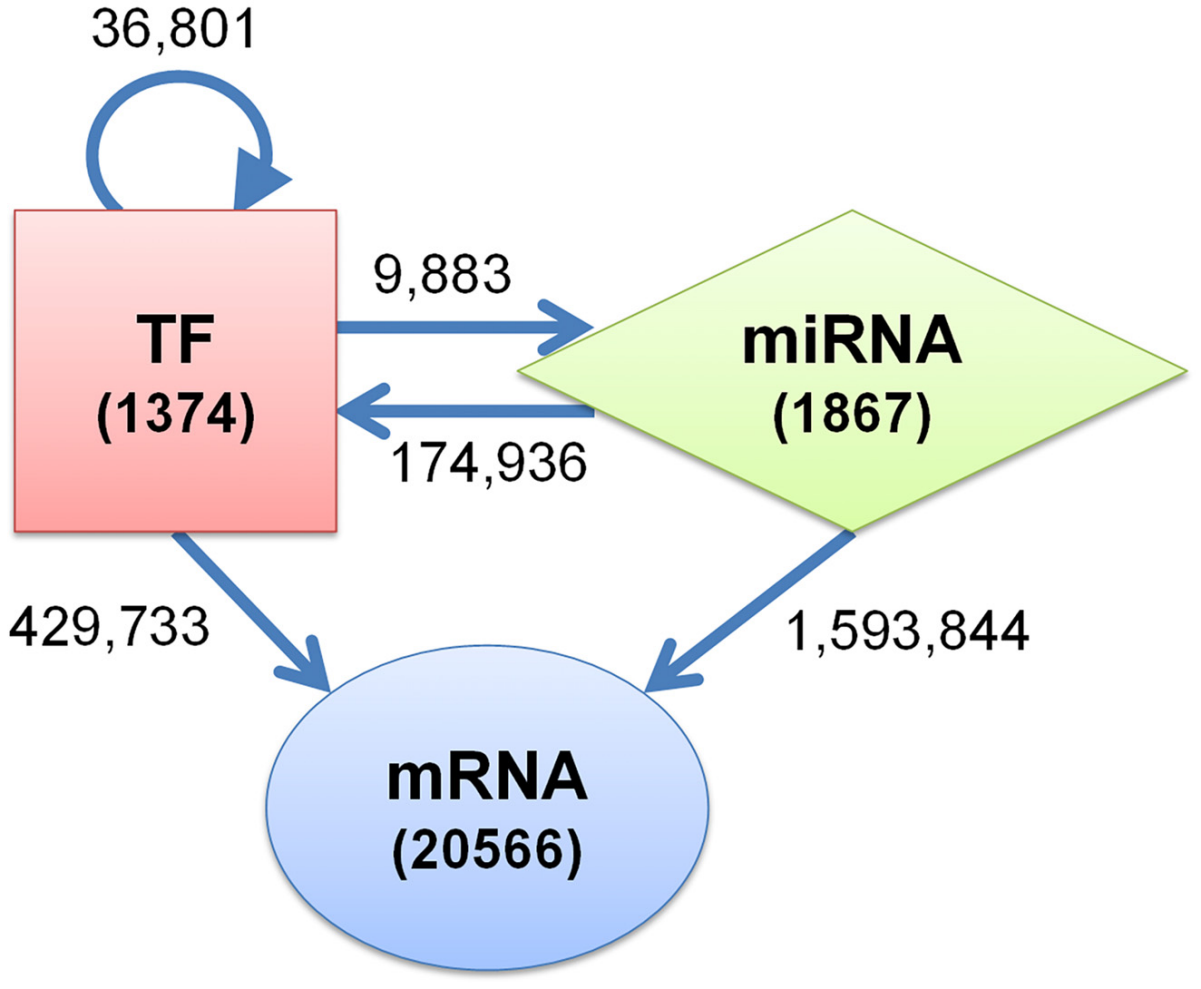
Integrated human transcriptional and post-transcriptional GRN. The network contains a total of 1867 miRNAs and 21,940 genes, including 1374 TFs and 20,556 non-TF genes (or mRNAs for simplicity). The numbers on the edges denote the total number of interactions between different types of nodes.

We constructed an integrated general regulatory network of all human genes and miRNAs using extensive experimental TF target data and *in silico* miRNA target data derived from public databases (Fig 1). The general human TF-miRNA-mRNA network presents a useful base for understanding the structural characteristics of human GRNs and deriving subnetworks involved in specific biological processes. For instance, in this study we derived the network of genes responsible for estrogen response of MCF-7 breast cancer cells as a subnetwork of the general network.

The nodes in our general human TF-miRNA-mRNA network represent 21,940 genes and 1,867 mature miRNAs based on gencode version 17 [23] and miRBase version 19 [24] annotations respectively. Out of 21,940 genes, 1,374 were marked as TFs and the remaining 20,566 were designated as non-regulatory genes which we call messenger RNAs (mRNAs) for simplicity. Genes were identified as TFs based on gene ontology annotation with term GO:0003700 “sequence-specific DNA binding transcription factor activity” and information obtained from TRANSFAC database [25].

The edges in the network comprised a total of 2,245,197 interactions between TFs, miRNAs and mRNAs. Out of the 1,374 TFs in the network, experimentally validated targets for 329 TFs were obtained from ENCODE and HTRIdb databases [26,27]. For the remaining 1,045 TFs no target information was included. Although regulatory interactions between genes are cell type specific, we ignored this specificity to include all known interactions in all cell types. There were in total 466,534 TF-target interactions including 36,801 TF-TF (including self-regulation), 429,733 TF-mRNA, and 9,883 TF-miRNA interactions. In addition, there were 1,768,780 miRNA-target interactions including 174,936 miRNA-TF and 1,593,844 miRNA-mRNA interactions. These miRNA targets were identified by combining information from four different *in silico* databases as described in the Methods section.

#### Node degree distributions

**Fig 2:**
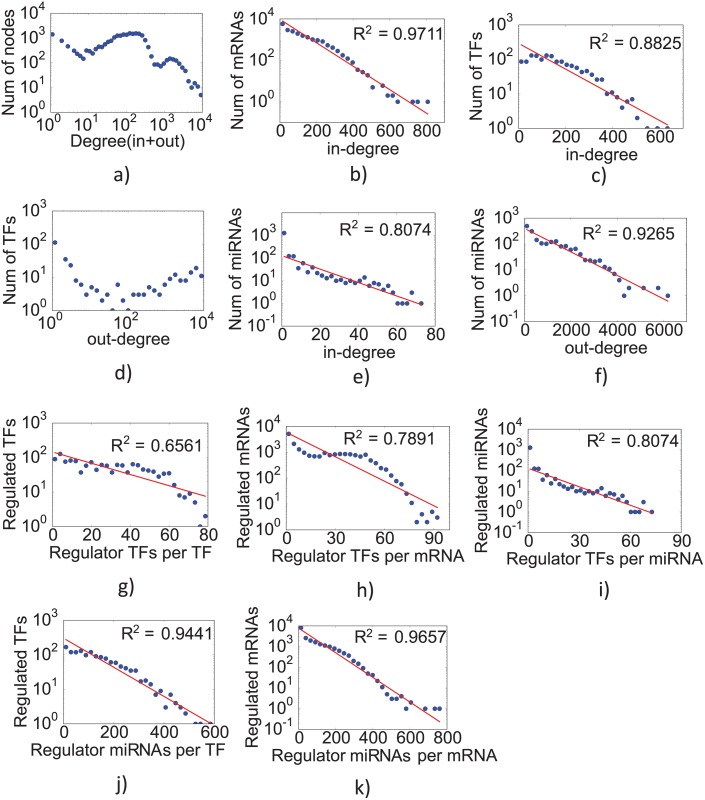
Degree distribution of the general human TF-miRNA-mRNA network over (a) all nodes, (b) mRNAs, (c,d) TFs (in, out degrees) and (e,f) miRNAs (in, out degrees), (g,h,i) regulation of TFs, mRNAs and miRNAs by TFs, (j,k) regulation of TFs and mRNAs by miRNAs.

To get a systematic understanding of the general human TF-miRNA-mRNA network, we examined the *degree distribution* of the network, *i*.*e*., the statistical distribution of the number of incoming and outgoing edges in a node. The overall network had a multimodal degree distribution (Fig 2a) which prompted us to separately analyse the degree distributions of different types of nodes and edges in the network (Fig 2b–2f). Most regulatory interactions were best fitted by an exponential distribution (r^2^ > 0.7; Fig 2b, 2c, 2e and 2f) implying that a molecule is more likely to regulate or be regulated by a few other molecules rather than many molecules simultaneously. A notable exception was the out-degree of TFs (Fig 2d), implying that TFs usually target a large number of genes (Table 1). Most mRNAs were regulated by several TFs and miRNAs (median 17 TFs, 76 miRNAs). TFs have a slightly larger number of regulating molecules (median 25 TFs, 111 miRNAs). Notably, miRNAs were usually regulated by a small number of TFs (median 3 TFs), whereas they in turn regulate a large number of TFs (median 74) and mRNAs (median 595.5). Furthermore, some TFs target a large number of molecules as indicated by high mean values of TF->mRNA (1377.35), TF->miRNA (83.75) and TF->TF (195.75) interactions although the corresponding median values are lower. Despite data incompleteness, these observations are in agreement with similar patterns reported in *C*. *elegans* gene network analysis [12].

**Table 1:** Statistics of degree distribution for various interactions in the integrated human GRN.

Interaction Type	Min	Max	Mean	Median
TF → TF	1	918	195.75	74
TF ← TF	1	80	27.06	25
TF → mRNA	1	11357	1377.35	3
mRNA ← TF	1	94	21.55	17
TF → miRNA	1	343	83.75	60.5
miRNA ← TF	1	74	9.39	3
miRNA → TF	1	595	99.45	74
TF ← miRNA	1	595	131.73	111
miRNA → mRNA	1	5741	893.41	595.5
mRNA ← miRNA	1	773	99.06	76

#### Node degree distributions correlate with gene functions and expression

We selected 500 nodes with highest in-degrees and 500 nodes with lowest in-degrees from the general human TF-miRNA-mRNA network for functional analysis. 500 genes is a reasonably large number to obtain statistical significance in gene set analysis [28] and represents 2.5 percent of the total number of nodes. Gene ontology analysis using DAVID [29] reported “transcriptional regulation” as the most enriched function for the 500 highest in-degree genes (139/500 genes, FDR 5.5e-11) and “nucleus” as their most prominent cellular compartment (205/500 genes, FDR 1.4e-19). In contrast, the 500 lowest in-degree genes were significantly enriched with “G-protein coupled receptors” (176/500 genes, FDR 6.9e-123) lying in the “cell membrane” (247/500 genes, FDR 2.0e-22) and participating in “cell surface receptor linked signal transduction” (203/500 genes, FDR 5.8e-73). The results were similar when the analysis was repeated on 1000 highest and lowest in-degree nodes.

**Fig 3:**
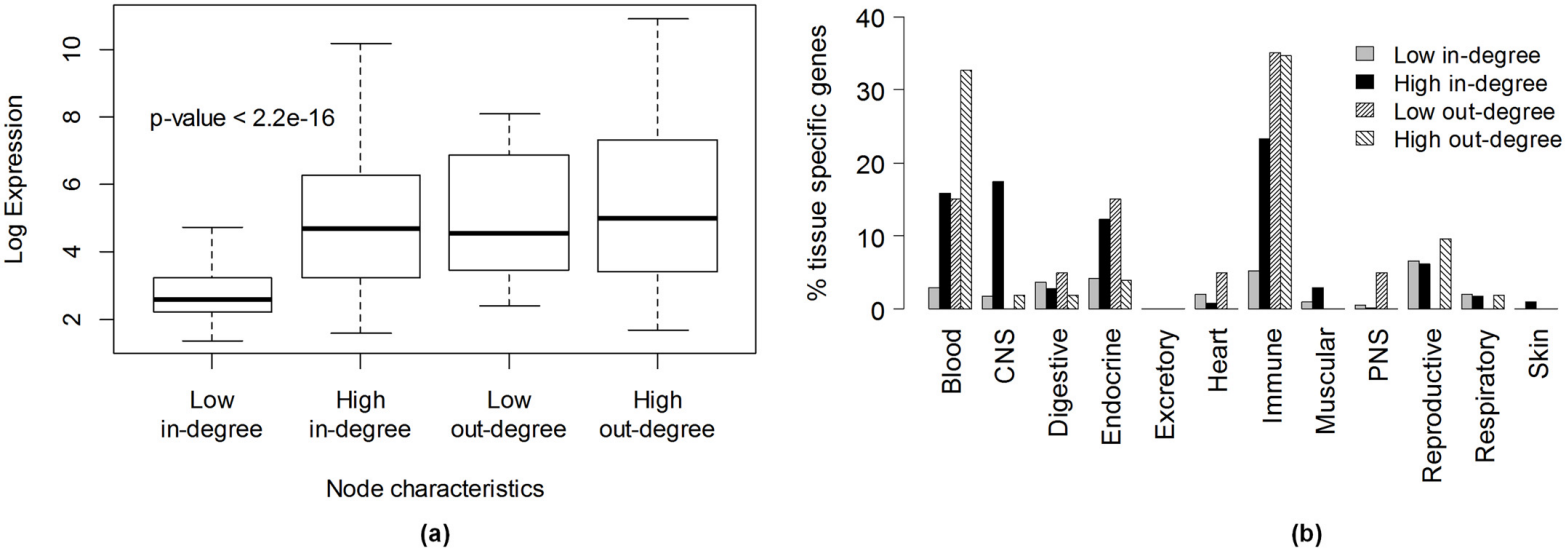
Relationship between a node’s in or out-degree in the integrated human TF-miRNA-mRNA network and its expression characteristics in BioGPS (a) absolute expression level, (b) tissue specific gene expression.

We used BioGPS [30,31] to study the expression profiles of these genes in 84 different human tissues. The low in-degree genes had a significantly lower absolute expression level across all tissues as compared to the high in-degree genes (Fig 3a). We also looked at tissue specificity of gene expression using a specificity measure (SPM) described by Xiao et al. [32]. The SPM of a gene in tissue *t* is a number in the range 0 to 1.0 calculated as $SPM_{t}=x_{t}/\sqrt{\sum_{t=1}^{T}x_{t}^{2}}$ where *x*_*t*_ is the gene’s expression level in tissue *t* and *T* is the total number of tissues. We chose *SPM*_*t*_>0.5 as indicative of tissue specificity which implies that the expression in tissue *t* outweighs the combined expression in all other tissues. Moreover, following Xiao et al. [32] we merged similar tissues into an integrative tissue (such as merging monocytes, B cells, T cells and peripheral blood in a single tissue called “Blood”) to account for the fact that spiking of gene expression occurs simultaneously in similar tissues. Thus the 84 tissues were squashed into 12 integrative tissues. 422 (84%) out of 500 high in-degree genes had tissue specific expression while in comparison only 150 (30%) out of 500 low-in-degree genes were tissue specific (Fig 3b), indicating that high node in-degree is associated with tissue specificity of gene expression.

We also analysed the BioGPS expression profiles of top 52 transcription factors which have an out-degree of more than 4000 and observed that 45 TFs (86%) were expressed in a tissue specific manner with 35 of them (67%) being highly expressed in the blood and the immune cells (Fig 3b).

#### Inferring the properties of complete human GRN by extrapolation

Our general human TF-miRNA-mRNA network contains only 10–15% of TF-target interactions expected in the complete network since out of more than 1300 TFs, only 120 TFs have been ChIPed in ENCODE and there is only limited target information for another 209 TFs in HTRIdB.

**Fig 4:**
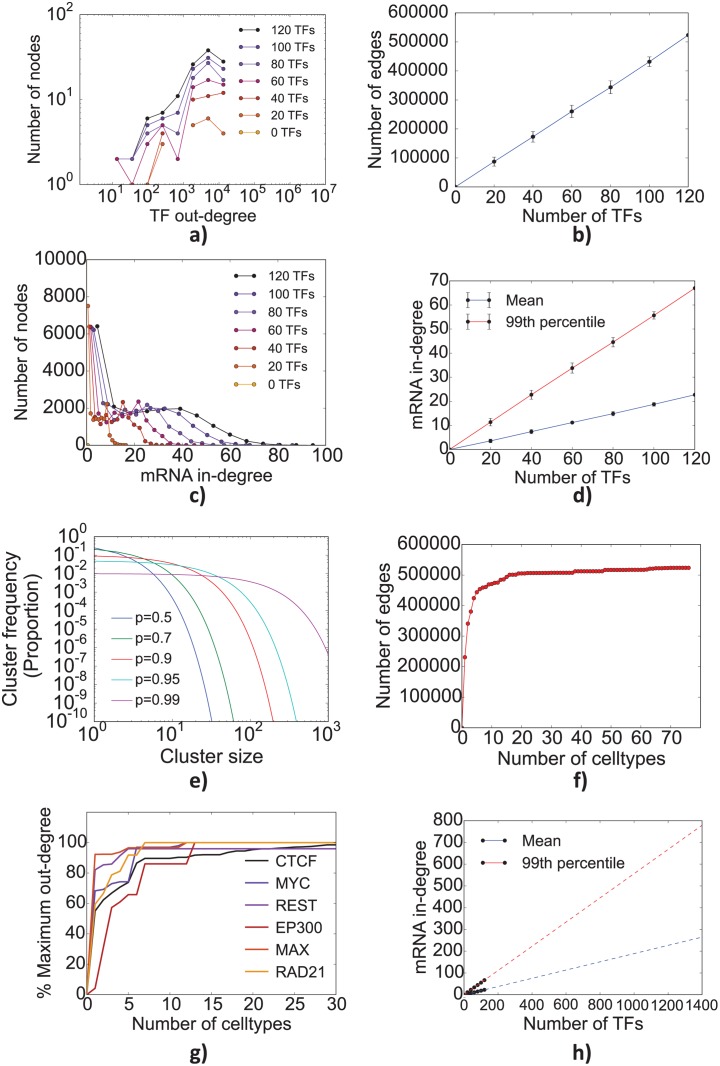
Evolution of the integrated human TF-miRNA-mRNA network with increasing number of ChIP’ed transcription factors and cell types in which transcription factors have been ChIP’ed. As more TFs are ChIP’ed, while the shape of TF out-degree distribution remains the same (a), a proportionate number of edges are added to the network (b). Addition of these new edges leads to a linear increase in the in-degrees of mRNA nodes both for average in-degree and high in-degree mRNAs (c,d). This is similar to percolation dynamics where the frequency of both average and large size clusters increases as an increasing number of lattice spaces are filled up (e). ChIP of the same TFs in more cell types adds fewer new edges to the network (f) and the TF nodes (g) with a plateau reached beyond 10 cell types. Shown in (h) is an extrapolation of mRNA in-degree if all known TFs in the human genome (~1400) were to be ChIP’ed.

We extrapolated the properties of the complete human GRN from the general trends observed in our partial network. We studied the evolution of network properties as ChIP experiments were progressively added. A ChIP experiment being added could be for a new TF, i.e., for which there is no existing target information in the GRN, or for an existing TF but in a new cell type so that there will be some additional outgoing edges for the TF. As ChIP experiments were added for new TFs the shape of the out-degree distribution of TFs did not change (Fig 4a) but the total number of edges in the network increased linearly (Fig 4b). As a consequence, the distribution of the in-degree of mRNA nodes and the location of the abrupt “drop” in number of nodes linearly increases with the number of TFs Fig 4c and 4d. The results suggest that as the number of TFs is increased, a fixed proportion of mRNAs in the GRN receive additional incoming edges resembling a network percolation like dynamics. Fig 4e shows a comparative plot of the theoretical cluster sizes obtained from random networks with successively higher probability of edges (denoted by *p*) between the nodes. Note that when a certain threshold number of TFs is reached, a giant cluster is expected to emerge within the network. In contrast, adding ChIP experiments for existing TFs contributed little to increase the number of edges in the network (Fig 4f) as the number of targets of a TF reached saturation after a handful of ChIP experiments (Fig 4g). From these trends we extrapolate that a complete integrated human GRN might contain ~5 million TF-target interactions. While a few hub nodes may have more than 750 incoming edges, a majority of genes might have approximately 250 incoming edges (Fig 4h).

### Benchmarking algorithms for identifying core regulatory genes

The general human TF-miRNA-mRNA network includes all known genes and interactions. However, the entire network of genes is not simultaneously active in a single cell. During a biological process certain genes are switched on (activated) and others are switched off (repressed) in order to produce the right repertoire of cellular products required in the process. Each biological process is governed by some core regulatory genes (TFs and miRNAs) that control the expression of a large number of downstream genes (mRNAs) to produce a specific repertoire of gene products. In this section we study gene networks active in specific biological processes and use network analysis algorithms for identifying their core regulatory genes.

#### Construction of MCF-7 gene network

We studied the network of genes that respond to estrogen treatment in the MCF-7 breast cancer cell line. After searching through public databases for appropriate human datasets where high quality gene expression, transcription factor binding and interventional data are available, we narrowed our interest to the MCF-7 cell line which is a model for hormone-responsive breast cancer [33]. Treatment of MCF-7 cells with 17-β estradiol (E2 or estrogen) drives the expression of thousands of genes mediated by the estrogen receptor (ER) and leads to extensive cell proliferation. We studied the network of genes that are differentially expressed in MCF-7 upon E2 treatment using four different public datasets present in NCBI’s Gene Expression Omnibus (GEO) reporting high throughput gene expression in hormone starved (Control) *vs*. estradiol treated (E2) MCF-7 cells (Table 2).

**Table 2:** Public MCF-7 gene expression datasets downloaded from NCBI’s Gene Expression Omnibus (GEO) database.

Category	GEO Series ID	Platform	Description	Reference
Datasets for building MCF-7 gene network	GSE11324	Affymetrix HG U133 plus 2.0	Control *vs*. 100nM E2 treatment for 12 hrs.	[34]
	GSE11352	Affymetrix HG U133 plus 2.0	Control *vs*. 10nM E2 treatment for 12 hrs.	[35]
	GSE42619	Agilent HG G4112F 4x44K	Control *vs*. 10nM E2 treatment for 24 hrs.	[36]
	GSE51403	Illumina HiSeq 2000 RNA-seq	Control *vs*. 10nM E2 treatment for 24 hrs.	[37]

**Fig 5:**
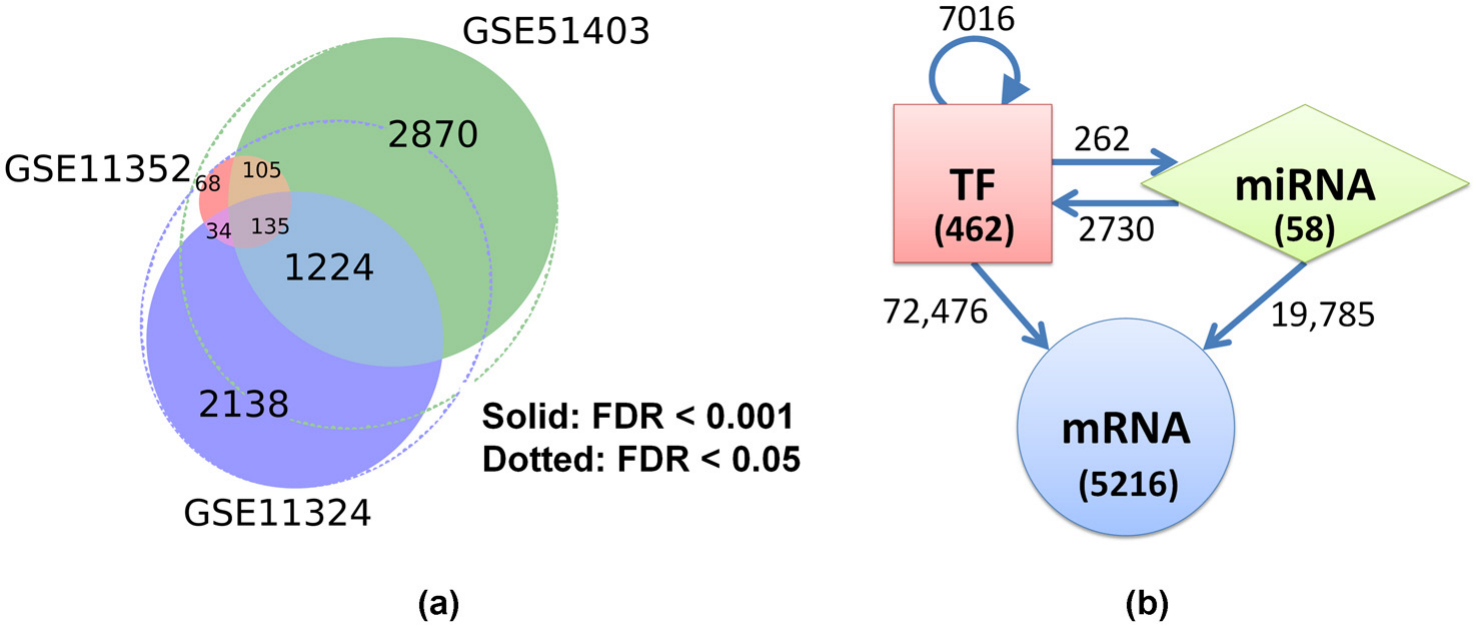
MCF-7 estrogen response gene network. (a) Differentially expressed genes with FDR < 0.001 were selected from two high quality datasets GSE11324 (microarray) and GSE51403 (RNA-seq). A significant number of genes were common between the two datasets, near about 70% if a FDR cutoff of 0.05 were to be used as shown by dotted ellipses. The union list between GSE11324 and GSE51403 was selected for network construction. The union list also had a good overlap with another dataset GSE11352. (b) The final MCF-7 network consisted of 5736 nodes including 462 TFs, 58 miRNAs and 5216 mRNA genes. The numbers on the edges denote the total number of interconnections between various types of nodes.

The treatment protocols were similar in all four datasets except for higher concentration of E2 used in GSE11324 and two different sample collection times of 12hrs (GSE11324, GSE11352) and 24hrs (GSE42619, GSE51403). In GSE11352 where both 12 and 24hr time points are reported, we observed that the list of differentially expressed genes (DEGs) at 12hrs was largely a subset of the list of DEGs at 24hrs and the directions of fold change were exactly the same between these two time points (see S1 Text). The technology platforms varied between two different microarrays (Affymetrix, Agilent) and RNA-seq (Illumina). Two experimental datasets, GSE11324 and GSE51403, showed higher sensitivity than the others, reporting 8,180 and 7,792 DEGs respectively at 0.05 FDR, and 3,362 and 4,094 DEGs respectively at 0.001 FDR (Fig 5a). These two experiments together included most of the DEGs found in the other two experiments (see S1 Text and Fig 5a). Hence we chose to use the union of DEGs in GSE11324 and GSE51403 at 0.001 FDR as starting DEG list. Although the overlap of gene lists between the two datasets was low (30%) at 0.001 FDR, relaxing the FDR to 0.05 in the comparison dataset increased the overlap to more than 60% (Fig 5a). Thus using a stringent FDR of 0.001 limited us to a higher quality DEG list. This list consisted of a total of 5,736 molecules including 462 TFs, 58 miRNAs and 5,216 mRNAs (Fig 5b).

Next, we derived the network of these 5,736 DEGs by taking this subset from our general human TF-miRNA-mRNA network. All 5,736 nodes and all the edges interconnecting them were considered. Out of the 462 TFs, only 106 TFs had one or more known targets in the network. The remaining 356 TFs either did not have any target information in ENCODE and HTRIdB or none of their known target genes were within the MCF-7 estrogen response network.

#### Identification of core regulatory molecules in MCF-7 response to estrogen

In order to identify the core regulators (TFs and miRNAs) in MCF-7 estrogen response GRN, we applied various strategies for ranking regulatory molecules (106 TFs and 58 miRNAs). Researchers usually look at most differentially expressed regulators or differentially expressed regulators with the most number of targets in the list of differentially expressed genes (i.e., regulators with maximum out-degree in the GRN) or regulators with maximum fold enrichment of differentially expressed (DE) targets in the data computed as [(#target genes that are DE)/(#DE genes)] / [(#target genes)/(#all genes)]. In addition, we used various network analysis based methods to rank the regulators, including maximum in-degree, centrality measures (closeness, betweenness), pagerank, and K-core. The top 20 ranked regulatory molecules identified by each strategy are shown in Table 3.

**Table 3:** Top 20 regulatory molecules identified by various ranking strategies in the MCF-7 estrogen response gene regulatory network.

Ranking Strategy	Top regulatory molecules (ranked 1 to 20 from left to right)
**Most differentially expressed regulators**	hsa-miR-941, GATA4, PGR, SIM1, CXCL12, hsa-miR-653, hsa-miR-489, EGR3, SOX3, HEY2, MYBL1, hsa-miR-548M, TMEM229A, FOXE3, FOXC2, CREB3L1, GRHL3, MYB, hsa-miR-623, hsa-miR-1231
**Maximum out-degree**	MYC, ELF1, TAF1, E2F6, E2F1, HDAC2, EGR1, CHD2, GATA2, USF1, RAD21, IRF1, GABPA, FOXA1, AR, SMC3, SRF, RFX5, GATA3, ZNF143
**Maximum target fold enrichment**	ESR1, STAT2, TP63, hsa-miR-4800-5P, GATA3, HIF1A, NFYA, TFAP2C, IRF1, SREBF1, GTF2F1, ATF3, BHLHE40, hsa-miR-4640-3P, RFX5, PBX3, SMARCC1, E2F1, USF2, FOXA1
**Maximum in-degree**	PURB, HNRNPD, NFAT5, ZBTB4, CHD2, STAT3, HMGB1, NAA15, ILF3, CREB1, FOXN2, NCOA3, GABPB1, HBP1, HIF1A, AFF4, ZNF143, ELK4, KLF10, ZNF367
**Maximum closeness centrality**	MYC, ELF1, TAF1, E2F6, E2F1, EGR1, HDAC2, CHD2, GATA2, RAD21, USF1, IRF1, AR, GABPA, FOXA1, SMC3, GATA3, YBX1, SRF, ZNF143
**Maximum betweenness centrality**	MYC, E2F6, ELF1, YBX1, E2F1, STAT3, GABPA, CHD2, EGR1, ESR1, TAF1, SRF, ZNF143, RAD21, FOS, ELK4, GATA2, AR, TFAP2C, JUNB
**Maximum pagerank**	MYC, MYB, ATF3, RUNX3, BRCA1, PPARA, ESR1, SMAD7, CXCL12, ASCL1, LMO2, CDKN1A, E2F1, TCERG1, SOX9, TP53, ETS2, SMAD3, TFAP2C, MEIS2
**Innermost K-core**	MYC, ELF1, TAF1, E2F6, E2F1, HDAC2, EGR1, CHD2, USF1, RAD21, IRF1, GABPA, SMC3, SRF, RFX5, GATA3, ZNF143, YBX1, USF2, STAT3

We used three different criteria to evaluate the effectiveness of various strategies towards identifying core regulators of the MCF-7 estrogen response GRN: (i) randomization test, (ii) literature evidence, and (iii) quantitative modeling of gene expression.

#### Randomization test for core regulators

We performed a randomization test to determine whether the core regulators of the MCF-7 estrogen response GRN identified by a ranking strategy were selected by chance. The randomization test evaluates the null hypothesis that the core regulatory molecules were selected due to the characteristics of the general TF-miRNA-mRNA network and not specifically for the MCF-7 ER network. In case the null hypothesis holds, further evaluation of whether the core regulators play an important role in regulating the MCF-7 estrogen response GRN will not be meaningful as the same set of core regulators will be reported by the ranking strategy for any biological process.

**Fig 6:**
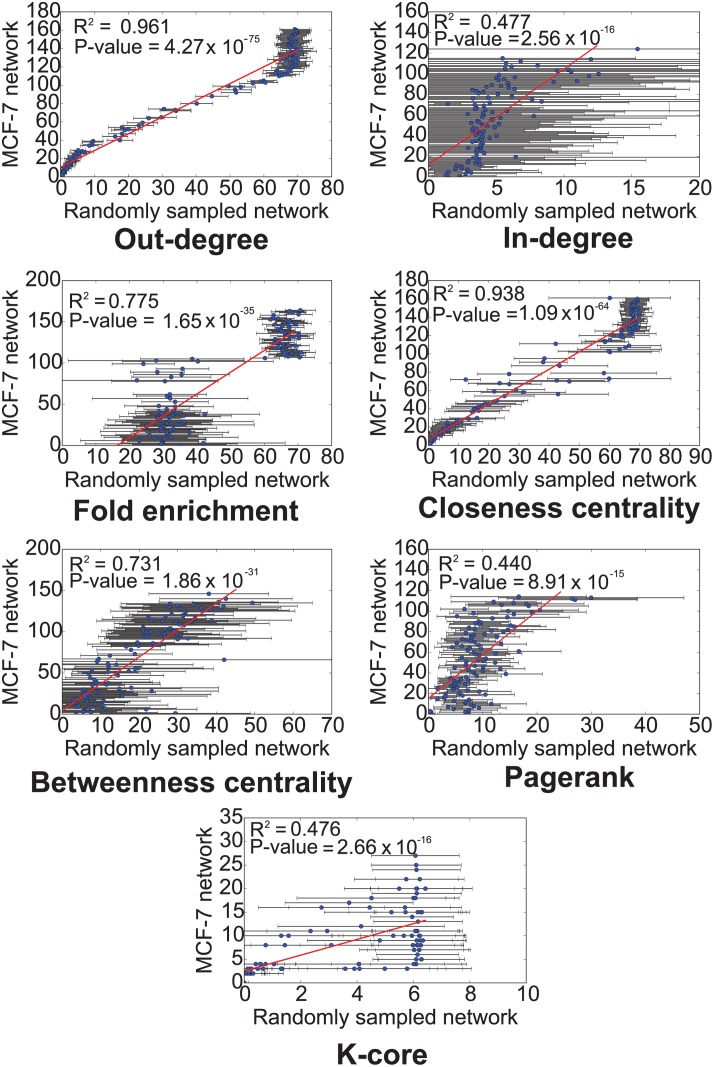
Randomization test to determine whether the core TFs and microRNAs identified in MCF-7 estrogen response GRN were obtained by chance. The scatter plot compares the core number of a TF in MCF-7 estrogen response GRN (y-axis) with its average core number over 10,000 randomly sampled networks (x-axis). To be comparable the randomly sampled networks contained the same number of TFs and miRNAs as the MCF-7 estrogen response GRN.

**Fig 7:**
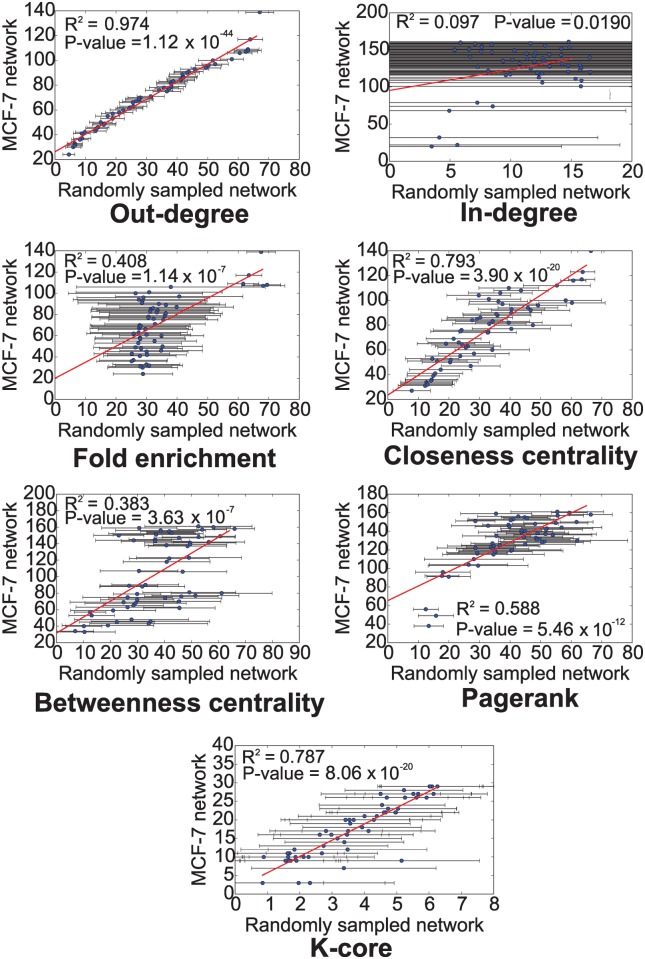
Randomization test to determine whether the core TFs and microRNAs identified in MCF-7 estrogen response GRN were obtained by chance. The scatter plot compares the core number of a microRNA in MCF-7 estrogen response GRN (y-axis) with its average core number over 10,000 randomly sampled networks (x-axis). To be comparable the randomly sampled networks contained the same number of TFs and miRNAs as the MCF-7 estrogen response GRN.

We generated 10,000 networks by randomly sampling nodes from the general TF-miRNA-mRNA network maintaining the same number of nodes in each randomly sampled network as the MCF-7 estrogen response GRN. During random sampling we also maintained the same proportion of TFs (462 of which 106 have known targets), miRNAs (58), and mRNAs (5216) as the MCF-7 estrogen response GRN. In each randomly sampled network we used the abovementioned strategies for ranking genes and checked the coefficient of determination *R* ^2^ (square of sample correlation coefficient) between the average rank of a regulator in a randomly sampled network (if it were present in a randomly sampled network) and its rank observed in the MCF-7 estrogen response GRN using the same strategy (Figs 6 and 7). Except for “most differentially expressed regulators”, all other strategies for ranking genes could be evaluated by this method. Ranking of regulators based on maximum out-degree and closeness centrality was very similar in MCF-7 estrogen response GRN and randomly sampled networks (*R* ^2^ > 0.9 in Figs 6 and 7). Rankings based on other strategies were mildly correlated. Thus the randomization test favored other ranking strategies over maximum out-degree and closeness centrality.

#### Literature validation of core regulators

We evaluated the biological relevance of core regulatory molecules identified by a ranking strategy to the MCF-7 estrogen response GRN by searching for evidence in the published literature. We used Google Scholar to quantify the number of publications in which a regulatory molecule has been cited in the context of estrogen stimulation of MCF-7 (see Methods). Based on this quantification we ranked all the regulatory molecules present in the MCF-7 ER network in descending order of the number of associated publications (see S1 Text for the full list). For instance, the gene ESR1 (Estrogen Receptor) was ranked 1^st^ with 84,700 associated publications. We then summed up the ranks of all top 20 regulatory molecules identified by a ranking strategy to produce its “literature validation score”, so that a lower scoring ranking strategy was considered better. The scores of various ranking strategies are shown in Table 4. In comparison, selection of any 20 regulators at random produced literature validation scores with a mean of 1349.8 and standard deviation 160.5 over 1000 random trials. The regulatory molecules identified using betweenness centrality and pagerank algorithms were found to have better literature evidence as compared to the other ranking strategies. Maximum out-degree and K-core measures also identified regulatory molecules that are well-known in the literature. However, regulatory molecules with maximum in-degree or that were most differentially expressed had poor literature validation.

**Table 4:** Literature validation scores for various ranking strategies (lower scores are better).

Ranking Strategy	Literature validation score
Most differentially expressed regulators	1563
Maximum out-degree	837
Maximum target fold enrichment	1165
Maximum in-degree	1584
Maximum closeness centrality	796
Maximum betweenness centrality	718
Maximum pagerank	728
Innermost K-core	894

#### Modeling of gene expression

**Fig 8:**
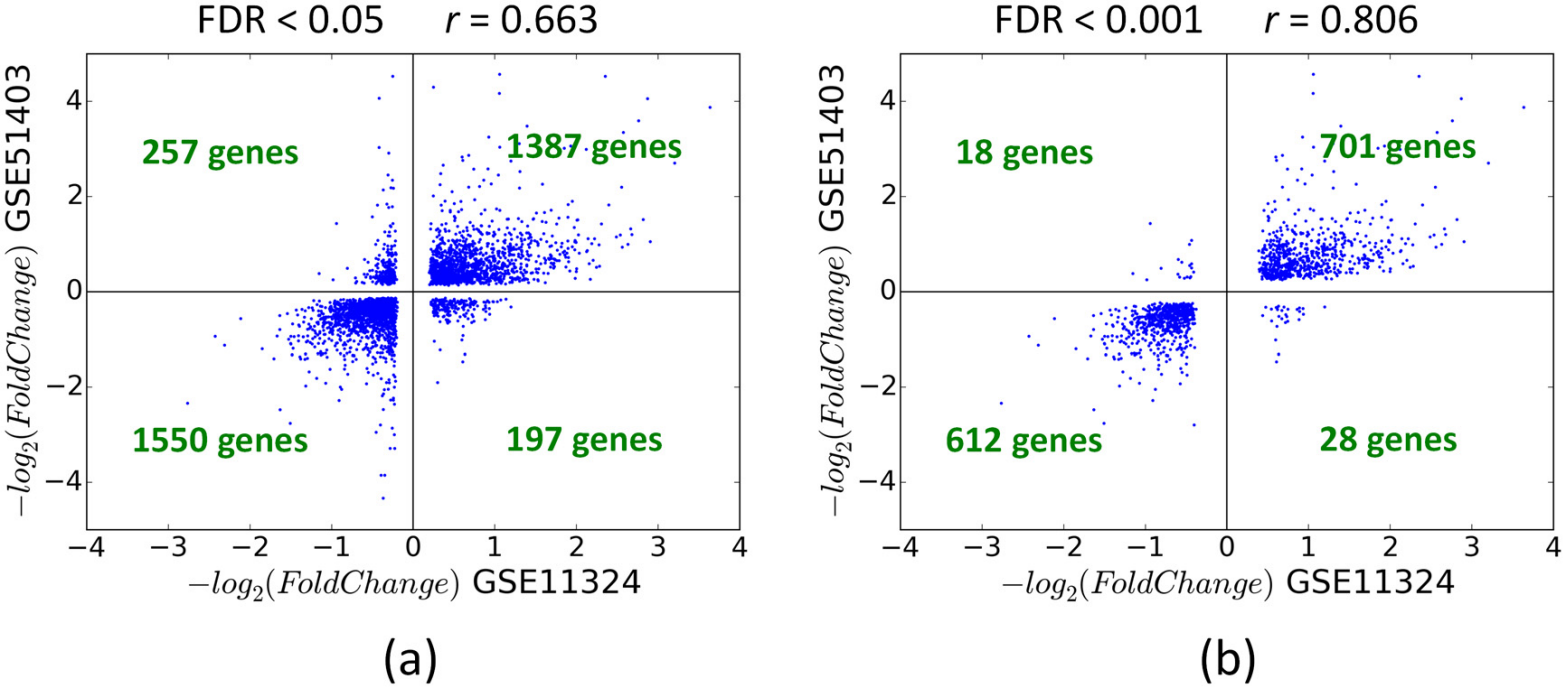
Comparison of gene expression measurements in two repeats of the same biological experiment. The scatter plot shows the measured fold changes of gene expression in E2 vs. Control treated MCF-7 cells in GSE11324 (x-axis) and GSE51304 (y-axis) experiments. Only genes which passed a FDR cutoff of 0.001 (a) or 0.05 (b) are shown. Although both datasets are of high quality, the absolute values of fold change are only moderately correlated. However, the direction of fold change is consistent for most of the genes.

We investigated whether the core regulators identified by a ranking strategy could explain the expression levels of remaining genes in the GRN. Recent studies on quantitative modeling of gene expression have attempted to model real valued log fold change values [38,39]. However, we observed a fair amount of biological variance in log fold change measurements. Between the two high quality gene expression datasets GSE11324 and GSE51403 used to derive our MCF-7 estrogen response GRN, the log fold change measurements were only a moderately correlated (Pearson coefficient *r* = 0.80 using FDR<0.001, and *r* = 0.663 using FDR<0.05) (Fig 8). However, the direction of gene expression was largely consistent in the two datasets (97% consistency using FDR<0.001, and 87% consistency using FDR<0.05). In order to minimize the effect of biological variation on prediction accuracy, we focused on binary classification of genes into up or down expressed categories as in some previous studies [9,40] instead of predicting real valued log fold changes.

We used the core regulators identified by a ranking strategy to model the expression of all other genes (called *target genes*) in a GRN. Only the edges directly connecting core regulators to target genes were considered in the model. Let there be *m* core regulators and *n* target genes. In the model a target gene *i*∈[1,…,*n*] is represented by an input-output pair (*X*_*i*_,*y*_*i*_) where $X_{i}=\left(x_{i}^{1},\ldots ,x_{i}^{m}\right)$ is a *m*-dimensional vector describing the regulation of the target gene by core regulators such that $x_{i}^{j}=1$ if there is an edge in the network connecting core regulator *j* to target gene *i*, or $x_{i}^{j}=0$ otherwise. The label *y*_*i*_ represents the expression level of target gene *i* as +1 if it is up expressed or -1 if down expressed. The classification model determines the closest possible approximation to the mapping *f*:**X**→*Y*. We used linear regression (LR) and support vector machines (support vector classification, SVC, and support vector regression, SVR) for supervised classification [38], and principal component analysis (PCA) for unsupervised classification [41]. An assumption built into our modeling strategy is that the core regulators act as mutually independent variables in controlling the expression of target genes. In LR and PCA models these variables combine additively (with a weight) whereas in SVC and SVR models they may combine nonlinearly such as multiplicatively.

Table 5 shows the classification accuracy obtained using core regulators identified by various ranking strategies using different classifiers. Classification accuracy was computed as area under the ROC curve (AUROC) or as Matthew’s correlation coefficient (MCC) averaged over a 5-fold cross validation procedure as described in Methods. AUROC value close to 0 (or MCC close to -1) indicates misclassification, AUROC = 0.5 (or MCC = 0) indicates random classification, and AUROC value close to 1.0 (or MCC close to 1.0) indicates perfect classification. Classification accuracies based on the core regulators selected by K-core, betweenness centrality, pagerank and maximum out-degree were comparable and consistently better than the other ranking strategies. The core regulators selected by K-core set into a SVR classifier reported the best classification accuracy of AUROC = 0.695 with the expression of slightly more than 70% of the genes correctly predicted in both up and down-regulated classes.

**Table 5:** Performance of explaining gene expression in E2 vs. control treated MCF-7 cells using core regulators identified by various ranking strategies. Three different mathematical or AI models were used for modeling gene expression: linear regression (LR), support vector machines (classification, SVC, and regression, SVR) and principal component analysis (PCA). Performance was measured as area under the ROC curve (AUROC) for real-valued estimators and using Matthew’s correlation coefficient (MCC) for binary classifiers in 5-fold cross validation.

Ranking Strategy	Area under ROC curve			MCC
	LR	SVR	PCA	SVC
Most differentially expressed regulators	0.567	0.575	0.572	0.149
Maximum out-degree	0.675	0.683	0.602	0.289
Maximum target fold enrichment	0.681	0.632	0.590	0.222
Maximum in-degree	0.586	0.585	0.586	0.155
Maximum closeness centrality	0.676	0.684	0.602	0.285
Maximum betweenness centrality	0.673	0.682	0.619	0.287
Maximum pagerank	0.653	0.646	0.620	0.254
Innermost K-core	0.674	0.695	0.605	0.294

We performed randomization tests to determine whether gene expression classification using network information was an artefact of machine learning algorithms. The null hypothesis is that the machine learning algorithm memorized the data so that network information did not play a role in classification results. Thus we replaced the inputs **X** which represent network information with randomly generated vectors of the same dimension while preserving the degree distribution of the network (using networkx function directed_configuration_model), and then performed gene expression classification with exactly the same procedure as before. In other words, we checked whether gene expression could be explained equally well using a random network. In 1000 trials of random networks, unsupervised classification using PCA yielded an average classification accuracy of AUROC = 0.501 with a standard deviation of 0.01, which is equivalent to a random predictor. Supervised classification using LR, SVR and SVC yielded a similar average classification accuracy of AUROC = 0.497 ± 0.02, AUROC = 0.504 ± 0.02 and MCC = -0.003 ± 0.01 respectively, leading to the conclusion that random networks are not explanatory of gene expression.

#### Comparative evaluation of ranking algorithms

In summary, among the ranking strategies benchmarked here, the core regulators identified using most differentially expressed or maximum in-degree criteria had comparatively poor validation against published literature and low performance in quantitative modeling of mRNA expression. The core regulators identified by maximum out-degree and closeness centrality strategies fared well in these two evaluations. However, in randomization tests they appeared to have been selected generally based on their prominence (by out-degree or closeness centrality) in the general TF-miRNA-mRNA network rather than specific relevance to the MCF-7 estrogen response GRN. The core regulators identified by betweenness centrality, pagerank and K-core fared well in all the three evaluation criteria. The core regulators identified by betweenness centrality and pagerank had better literature evidence as compared to the K-core regulators, including important genes such as estrogen receptor (ESR1) and androgen receptor (AR), which are central to the estrogen response. In contrast, the K-core regulators were best suited for quantitative modeling of gene expression.

### Hierarchical organization of MCF-7 estrogen response GRN using K-core

#### K-core structure of MCF-7 estrogen response GRN

**Fig 9:**
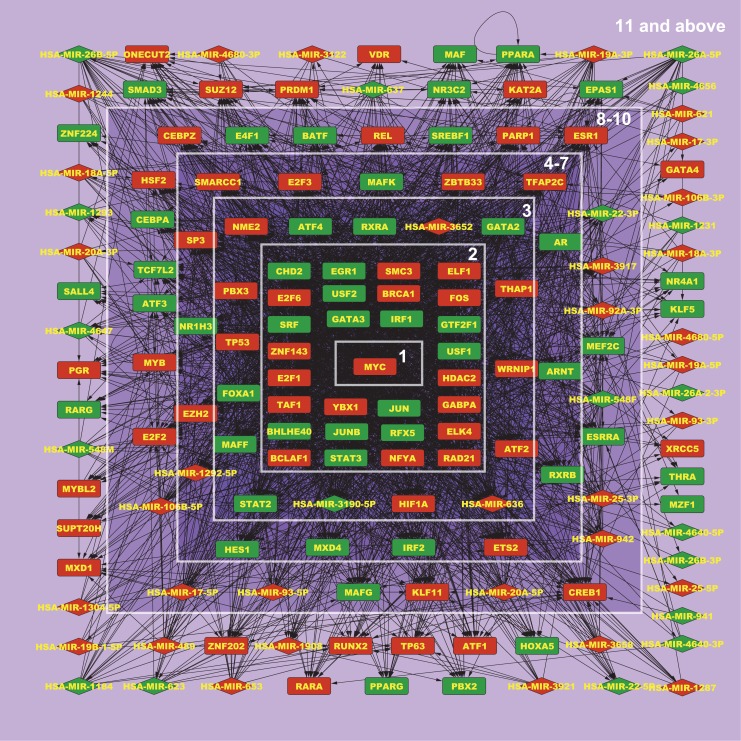
Hierarchical organization of all regulatory molecules, including 106TFs and 58miRNAs, in MCF-7 estrogen response GRN using K-core algorithm. TF and miRNA nodes are represented by rectangles and diamonds respectively. Nodes are colored red or green depending upon whether the molecule’s expression is up or down-regulated in E2 vs. Control cells. The hierarchy is based on the principle of network centrality where nodes which are more important for the flow of regulatory information are more towards the core. Nodes in core 1 (Myc) are most central, followed by nodes in cores 2, 3, and so on in decreasing order of centrality. Some cores have been clubbed together for ease of visualization.

Apart from identifying core regulatory molecules it is also important to organize a GRN in a structure that helps in understanding the flow of regulatory information. Following previous studies on yeast and bacterial GRNs [42,43], we used the K-core algorithm to organize the regulatory molecules in MCF-7 estrogen response GRN in a layered hierarchy. The K-core algorithm is generally used for identifying a set of *central* or *K-core* nodes in a network all of which have a degree of at least K. It works by iteratively removing leaf nodes which have degree less than K (all nodes with degree one are removed in the first iteration, nodes with degree two are removed in the second iteration, and so forth) so that in the final or K’th iteration only the K-core set of nodes remains. Each node in the network is assigned a *core number* based on the iteration during which it was removed by K-core. The K-core nodes are well interconnected with each other as well as with the rest of the nodes in the network and thus most of the information flow in a network takes place through the K-core nodes [44,45]. In the MCF-7 estrogen response GRN, 29 iterations of K-core were required until the final core was obtained and it consisted of only one molecule, the transcription factor Myc, a well-known oncogene. We then ranked all the regulatory molecules from 1 to 29 in the opposite order of their core number, i.e., the iteration in which they were removed by K-core. This resulted in a hierarchical organization of all the molecules with Myc, having rank 1 in the innermost core, followed by other molecules (Fig 9).

Importantly the core number obtained using the K-core algorithm has no relationship with the cascade of gene expression that actually takes place in the biological system. For instance, the transcription factor estrogen receptor (ER), which is encoded by the gene ESR1, is the key TF responsible for estrogen response in MCF-7 cells. Upon estrogen stimulation, ER moves to the cell nucleus and activates the transcription of a number of important TFs (such as Myc), triggering a cascade of gene expression which ultimately results in the activation of thousands of genes. However, in K-core analysis the gene ESR1 was found in the 8^th^ core. This is because ER is not central to information flow in the network as it has fewer direct targets compared to some of the other TFs that it regulates. On the other hand Myc is at the center of the core since it directly influences a large number of other TFs and miRNAs. As shown above (in the section titled “literature validation of core regulators”) we confirmed that the molecules placed within the first three cores are known in the literature to have key roles in the estrogen response of MCF-7 cells.

#### Nodes in the inner cores are more explanatory of gene expression

In order to test the hypothesis that the layered hierarchy produced by K-core places more influential or core regulatory molecules in the inner cores, we studied the contribution of regulators in various cores of the MCF-7 estrogen response GRN towards explaining the expression levels of mRNAs. We repeated the quantitative gene expression classification experiments as described above using regulatory nodes selected with varying cutoffs of the core number, K. Initially, several regulatory nodes were selected with a loose criterion of K ≤ 15. Subsequently, the number of regulatory nodes was reduced by progressively decreasing the cutoff for K until a minimum of K ≤ 2, which considered only the innermost core regulators. The overall classification accuracy decreased slightly as the number of regulatory nodes was gradually reduced from 111 (K ≤ 15) to 29 (K ≤ 2): AUROC for LR decreased from 0.733 to 0.687, AUROC for SVR decreased from 0.742 to 0.693, and MCC for SVC decreased from 0.364 to 0.300 (Table 6). The inner and outer core regulators were also used separately for gene expression classification in order to estimate their individual contributions (bottom half of Table 6). Classification accuracy was highest (LR AUROC = 0.687, SVR AUROC = 0.693, SVC MCC = 0.300) using 29 innermost core regulators (K ≤ 2), lower (LR AUROC = 0.535, SVR AUROC = 0.580, SVC MCC = 0.130) for the next outer set of 16 regulators (K = 3), and further lower for subsequent sets of outer regulators. Thus, the inner K-core regulators were found to be more explanatory of gene expression than the outer ones.

**Table 6:** Gene expression classification in the MCF-7 estrogen response GRN using various selections of regulatory nodes based on their core numbers, K, in K-core hierarchy. In the top half of the table the innermost core regulators (K ≤ 2) are always included and the cumulative effect of adding further core regulators is measured. In the bottom half of the table the innermost core regulators (K ≤ 2) are excluded in order to measure the individual contributions of regulators at various core levels. Classification accuracy is reported in terms of area under the ROC curve (AUROC) for real valued classifiers (LR, SVR and PCA) and Matthew’s correlation coefficient (MCC) for binary classifiers (SVC).

Selected regulators			Area under ROC curve			MCC
K cutoff	# Regulators	# Targets	LR	PCA	SVR	SVC
K ≤ 15	111	5625	0.733	0.609	0.742	0.364
K ≤ 10	89	5647	0.715	0.608	0.725	0.349
K ≤ 7	61	5675	0.696	0.606	0.703	0.309
K ≤ 3	45	5691	0.692	0.606	0.699	0.311
K ≤ 2	29	5707	0.687	0.610	0.693	0.300
K = 3	16	5720	0.584	0.535	0.580	0.130
3 < K ≤ 7	16	5720	0.570	0.525	0.565	0.084
7 < K ≤ 10	28	5708	0.569	0.525	0.568	0.091
10 < K ≤ 15	22	5714	0.569	0.572	0.609	0.165

#### Nodes in the inner cores are biologically more relevant

As described above and in the Methods section, we measured the biological relevance of individual genes by ranking them according to their number of Google scholar citations in the context of MCF-7 estrogen response. Then we computed the average rank of genes in each core to assess the overall biological relevance of a core. Lower average rank indicates greater known relevance to MCF-7 estrogen response biology. The average rank was lowest for the innermost cores (K≤2) and increased for each of the successive outer cores (Table 7) indicating that the regulators in the inner cores are biologically more relevant than those in the outer cores to the estrogen response of MCF-7 cells.

**Table 7:** Literature validation in terms of the average rank of genes in various cores of the K-core hierarchical organization of MCF-7 estrogen response GRN (lower scores are better).

Core(s)	# Regulators	Average rank
K≤2	29	47.8
K = 3	16	52.4
4≤K≤7	16	56.8
8≤K≤9	16	78.1
K = 10	12	80.4

#### Contribution of miRNAs in explaining gene expression

miRNAs are fine regulators of gene expression. We tested the regulatory contribution of miRNAs by measuring the accuracy of gene expression classification with and without including miRNAs within the list of regulators. In the MCF-7 estrogen response GRN most of the miRNAs occupied the outer cores in the hierarchical organization produced by K-core while none of them was within the innermost core (K ≤ 2), supporting the conclusion that miRNAs are not among the most important molecules that direct information flow in a gene network (Fig 9). Classification accuracy dropped slightly when miRNAs were excluded (Table 8) indicating a minor influence of miRNAs in regulating gene expression in the present network. A possible reason is that we have used *in silico* miRNA target information in this study which is generally considered inferior to experimental target information.

**Table 8:** Performance of gene expression classification in the MCF-7 estrogen response GRN with and without the inclusion of miRNAs in the list of regulators. Each row of the table represents a different selection of regulatory nodes based on their core number, K, in the hierarchy produced by K-core. Classification accuracy is reported in terms of the area under the ROC curve (AUROC) for LR and SVR.

Selected regulators	With miRNAs (AUROC)		Without miRNAs (AUROC)	
K cutoff	LR	SVR	LR	SVR
K ≤ 15	0.733	0.742	0.695	0.705
K ≤ 10	0.715	0.725	0.695	0.705
K ≤ 7	0.696	0.703	0.694	0.700
K ≤ 3	0.692	0.699	0.689	0.696

## Discussion

We described the construction of human TF-miRNA-mRNA networks using experimental TF-target and *in silico* miRNA-target information from public databases and algorithms for identifying their core regulatory genes and organizing them into a meaningful hierarchy.

Apart from its methodological contributions this study has also given some interesting insights into the topology of human GRNs. While most TFs tend to have high out-degrees, most mRNAs have low to medium in-degrees. There are only few hub mRNAs that attract many edges into them. This implies that there are many small cohorts of TFs that regulate various mRNAs. Usually a cohort of TFs regulates genes in a specific biological process. Thus could be many different cohorts of TFs regulating many varieties of biological processes. We observed that mRNAs with low in-degrees had low absolute expression levels and low tissue specificity and were usually present outside the nucleus performing housekeeping or signal transduction type of functions. On the other hand mRNAs with high in-degrees were associated with high expression level, high tissue specificity and were present in the nucleus taking part in transcriptional activity and other related functions in context of DNA. The association between high in-degree and tissue specificity makes biological sense as more number of TFs required for regulating a gene will allow greater fine tuning of its expression.

Although our current network is incomplete with only 10–15% of the estimated number of TF-target interactions known, by extrapolation we attempted to describe the characteristics of a full TF-miRNA-mRNA network. A full network is estimated to contain ~5 million TF-target interactions with an average mRNA in-degree of 250 and a few hubs with up to 750 incoming edges. Assuming that a TFBS is on average 8 bp long and a TF has on average 5 binding sites per gene (these values are rough estimates based on ENCODE data), the total regulatory DNA would span 2 billion bp. This estimate is on the higher side as we have neglected overlapping or competing TFBS and we have made a generous estimate of the total number of TF-target interactions. However, a large chunk of human DNA could carry regulatory signals.

The fact that betweenness centrality, pagerank and K-core algorithms performed the best in identifying core regulators of a GRN implies that nodes with higher and more direct connectivity with other nodes are more influential or core to biological processes. This is tied in to the hierarchical organization of GRNs. The concept of hierarchical organization in GRNs has been in place for many years. Earlier uses of terms such as “master regulators” [46] implied a pyramidal control structure similar to social organizations where few and influential individuals at the top of the hierarchy supervise a larger group of individuals below them. Initial studies of GRNs in simpler organisms such as *E*. *coli* and yeasts supported this idea [47,48]. However, the notion of hierarchy and master regulators has undergone changes as our understanding of transcriptional networks has evolved [49]. Regulation of gene expression is now understood to lie somewhere in between pyramidal “autocracy” (master regulators influencing thousands of genes) and flat “democracy” (all genes exerting regulatory influence on all other genes) [50]. For instance, Jothi et al. [20] used a vertex sort algorithm to organize yeast transcription factors in a three-layered hierarchy (top, core and bottom) having a feed-forward structure in which the top layer TFs regulate the core and the bottom layer TFs, the core layer TFs regulate the bottom layer, and the TFs in a layer regulate each other. Currently the study of modular structure of GRNs is gaining interest where groups of genes co-regulate and coordinate specific functions [51,52].

Our K-core based hierarchical organization implies a notion of *network centrality* or the relative influence or importance of nodes in a network. Nodes found in the inner cores are considered to be more influential or *central* than those in the outer cores. Although there are many measures of centrality [53], our choice of K-core centrality was motivated by the virtue of its being a combination of both global and local properties of a node as it lies somewhere between degree centrality which identifies nodes that are globally hubs, and betweenness centrality which identifies nodes that are locally important for information flow in a network [54]. In protein interaction networks, essential and evolutionary conserved proteins were found towards the innermost cores [55]. In internet networks, nodes with better routing capabilities, *i*.*e*. which can choose several paths to connect to another node, were in the inner cores [56]. In social networks and in spread of infectious diseases the most efficient spreaders were those located within the core of the network as identified by K-core [44,45,57]. It appears that in our hierarchical organization regulators central to the flow of regulatory information are in the innermost cores as they are most explanatory of gene expression. Previously it has been shown that greater number of TF-mRNA interactions is associated with more number of cores in the hierarchical organization of a GRN [42]. We observed a total of 29 cores in the K-core organization of MCF-7 estrogen response GRN as compared to between 6–9 cores observed in yeast GRNs [42], which agrees with the observation that human GRNs have higher degree of interconnectivity between genes than yeast GRNs.

### Sources of error

While our knowledge of bacterial and yeast GRNs is fairly comprehensive, human GRNs have only been partially reconstructed. Recently, a core human GRN interconnecting 475 TFs was reported separately for 41 different tissue types [58] by combining *in silico* predicted TF binding sites and *in vivo* DNaseI footprints. A human GRN was also reconstructed from ENCODE data [13]. In the present study we reconstructed a network of human genes and microRNAs using experimental TF target and *in silico* predicted miRNA target information. The TF target information obtained from ENCODE and HTRIdB covered only 329 out of 1374 TF nodes in our network and hence target information for more than two-thirds of the TFs was missing. Our analysis based on an incomplete network is prone to errors. Furthermore, TF target information obtained from ENCODE and HTRIdB is not tissue specific and hence it is only approximately correct when used for a specific tissue or cell line. Another source of error in our network reconstruction is that biochemical binding of a TF in the promoter region of a gene does not necessarily translate to a functional interaction between the TF and the target gene. Functional interaction requires additional criteria to be fulfilled such as the position of TF binding relative to the transcription start site and the context of other TF binding sites in its vicinity. Some proprietary databases contain information of functional interaction between TFs and their target genes compiled from published literature. This information can in fact be more accurate for delineating true interactions between TFs and their target genes. Despite all of these limitations, our network was able to explain gene expression with up to 70% accuracy (MCC = 0.4), which was significant against randomization tests. We are optimistic that prediction accuracy can be substantially improved as more extensive and accurate data of TF targets becomes available in the near future.

Due to lack of experimental data on miRNA targets, we obtained this information from a combination of four different publically available *in silico* miRNA target prediction tools, each of which has a different underlying methodology. A miRNA target was considered valid only if supported by at least two different tools (we also separately tried considering only targets supported by at least three different tools and observed similar results). We observed in our randomization tests that *in silico* miRNA target information contributed little towards explaining gene expression. Thus experimental data on miRNA targets would add important information to the network.

### Conclusion

In conclusion, this study presents an optimistic view of the usefulness of static gene networks for identifying core regulators within a densely interconnected system of genes and explaining gene expression.

## Methods

### Transcriptional regulation data

Regulatory targets of transcription factors (TFs) were identified using two public databases—Encode and HTRIdb. High throughput transcription factor binding profiles obtained from 423 ChIP Seq experiments on 76 different human cell types were downloaded from the ENCODE consortium website [27,59]. The ChIP Seq experiments reported a total of 5.8 million normalized peaks of 120 unique transcription factors in the human genome build 37 (hg19) reference assembly. The annotation of human genes and miRNAs in the same reference assembly was downloaded from the Gencode website (version 17) [23]. Following previous studies [12,39], any gene or miRNA whose TSS falls within ± 1kb of a transcription factor binding peak was considered as direct target of the transcription factor. Restricting or expanding the definition of target gene from ±0.25 kb to ±4kb had little effect on the number of TF-target or miRNA interactions (see S1 Text). Thus a total of 428,769 unique TF-gene interactions and 9,883 unique TF-miRNA interactions were identified. Furthermore, data of experimentally verified targets of 284 unique TFs totaling 51,871 TF-gene interactions, was downloaded from the HTRIdb database [26]. Combined together the Encode and HTRIdb data contributed a total of 466,534 unique TF-gene interactions for 329 unique TFs.

### Post-transcriptional regulation data

**Fig 10:**
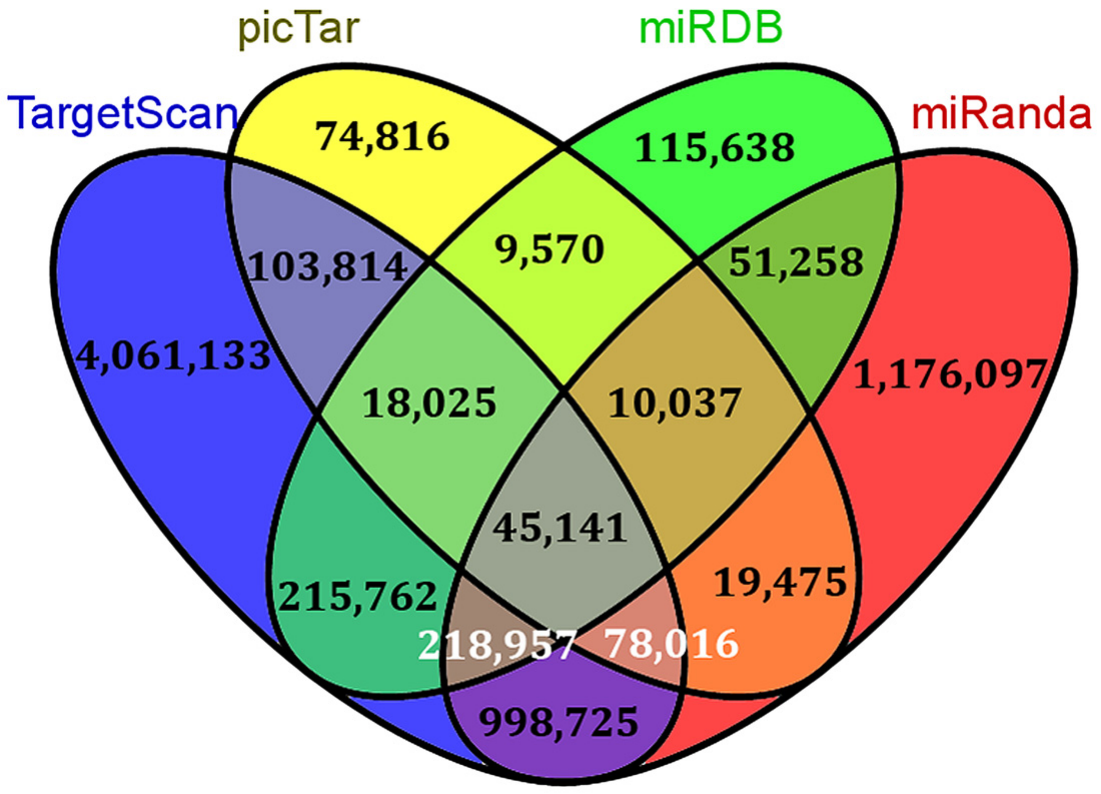
Overlap of miRNA-mRNA interactions predicted *in silico* by various tools.

*In silico* predicted miRNA-mRNA interactions were downloaded from four different databases, including miRanda [60], TargetScan [61], picTar [62] and miRDB [63]. Each of the above tools uses a different methodology for predicting miRNA targets. Interactions which were found in at least two out of four databases were chosen in the present study, which summed up to 1,768,780 miRNA-mRNA interactions as shown in Fig 10.

### Microarray data analysis

All public microarray gene expression datasets were downloaded from NCBI’s Gene Expression Omnibus (GEO) [64] in the GSE series matrix format. The series matrix files were analyzed using the Bioconductor package *limma* [65] in R statistical language to obtain differential gene expression.

### Network analysis

Network analysis, including network construction, subsampling and computation of centrality measures for nodes was performed using version 1.9.1 of *networkx* library [66] in Python programming language. All networks were constructed as directed networks using DiGraph class of *networkx*. Definitions of centrality measures used in this study (out_degree_centrality, in_degree_centrality, closeness centrality, betweenness centrality, pagerank, core_number) can be accessed via *networkx* documentation:

http://networkx.github.io/documentation/networkx-1.9.1/reference/algorithms.centrality.html

http://networkx.github.io/documentation/networkx-1.9.1/reference/algorithms.link_analysis.html

http://networkx.github.io/documentation/networkx-1.9.1/reference/algorithms.core.html

### Literature validation of core regulators

We used Google Scholar to perform a query using the format ‘+“MCF-7” +estrogen +*regulator’* where *regulator* is the gene symbol of the regulatory molecule whose biological relevance to the MCF-7 ER network is being evaluated. The number of results (publications) returned by Google Scholar was taken as a quantitative measure of the relevance of a molecule to the MCF-7 ER network. Since the same molecule may referred by different symbols in the literature, we downloaded all synonyms of a gene symbol from the HGNC website (http://www.genenames.org/cgi-bin/download) and took the maximum number of results returned by any of the synonyms as the final number. For some genes such as AR, the gene symbol was too short to be meaningfully queried. In such cases we queried by the full name of the molecule, such as +“Androgen receptor” in this case.

### Quantitative modeling of gene expression

Quantitative modeling was used to explain the expression status of genes in a GRN as either up or down expressed based on the core regulators (TFs and miRNAs) that target them in the network. Let there be *m* core regulators and *n* target genes. In the model the target genes were represented by input-output pairs (*X*_*i*_,*y*_*i*_), *i*∈[1,…,*n*] where $X_{i}=\left(x_{i}^{1},\ldots ,x_{i}^{m}\right)$ is a *m*-dimensional vector such that $x_{i}^{j}=1$ if there is an edge connecting core regulator *j*∈[1,…,*m*] to the target gene *i*, or otherwise $x_{i}^{j}=0$, and *y*_*i*_ = +1 if target gene *i* is up expressed or *y*_*i*_ = −1 if it is down expressed. We used various mathematical models to approximate target gene expression, *Y*, as a function of regulatory inputs, **X**, *viz*., linear regression (LR), support vector machines (support vector classification, SVC, and support vector regression, SVR) and principal component analysis (PCA), which are individually described below.

We set up a K-fold validation procedure where the total set of *n* target genes was randomly subdivided into K subsets of equal sizes. K iterations of classification were performed where in each iteration K-1 subsets of genes were used for training a mathematical model while the one remaining subset was used for testing its classification performance. Over the K iterations, each gene subset was used exactly once as test set for assessing the performance of the mathematical model. The classification performance computed as area under the ROC curve (AUROC) was reported as its average over the K iterations. In our experiments we performed a 5-fold cross validation.

Linear Regression (LR) was used to model the expression of target genes, *y*_*i*_, as

$$y_{i}=\beta_{0}+\sum_{j}\beta_{j}x_{i}^{j}+\varepsilon_{i},$$

where *β*_0_ is the intercept term representing basal expression level of all genes, *ε*_*i*_ is the error term, and *β*_*j*_ are the weights representing the relative influences of core regulators *j* in controlling the expression of target genes. The weights and intercept were analytically determined by the formula $\hat{\beta }={\left(X^{T}X\right)}^{-1}X^{T}Y$. The fitted expression levels of the genes ${\hat{y}}_{i}=y_{i}-\varepsilon_{i}$ were computed as $\hat{Y}=X\hat{\beta }$.

### Principal component analysis

The principal components of the input matrix **X** were computed by determining the eigenvalues, {*λ*_1_,*λ*_2_,…,*λ*_*m*_}, and eigenvectors, {*v*_1_,*v*_2_,…,*v*_*m*_}, of its covariance matrix, **XX** ^*T*^, satisfying the condition

$$\left(XX^{T}\right)v_{j}=\lambda_{j}v_{j},$$

where *λ*_*j*_ are scalars, and *v*_*j*_ are *n*×1 dimensional column vectors. The normalized eigenvectors ${\bar{v}}_{j}=v_{j}/\left\|v_{j}\right\|$ were called the principal components of **X**. The principal components were ordered such that the largest eigenvalue corresponds to the first principal component and the smallest eigenvalue corresponds to the last (*m* ^th^) principal component. The first principal component was used as a predictor for target gene expression, i.e., $\hat{Y}=v_{1}$.

Support vector machine was provided the *m*-dimensional rows of input matrix **X** as input vectors and the corresponding gene expression class, *Y*, as binary (+1 or -1) outputs. We used the libSVM software library [67] accessed via its in-built Python wrappers to implement both classification (SVC) and regression (SVR) models. In both models we used the radial basis function (RBF) kernel. The parameter γ of the kernel and the cost (or regularization) parameter *C* of the SVM model were chosen by optimization using grid search [68], but the cross-validation procedure was done differently in this case to avoid overfitting the parameters. We separated the data into 80% training and 20% held out test data. On the 80% training data we performed 5-fold cross validation while using grid search to select the best classification accuracy. Then the chosen parameters were used to train a SVM model on the entire 80% training data. The accuracy of this model was tested on the held out test data. The training data and held out test data were rotated in a 5-fold cross validation to obtain the average classification accuracy.

### Computation of area under the ROC curve (AUROC)

The LR, PCA and SVR models produced real valued estimates, ${\hat{y}}_{i}$, of the target gene expression levels, *y*_*i*_. The real valued ${\hat{y}}_{i}$ were converted to binary class predictions by selecting a discrimination threshold *t* such that $y_{i,predicted}=\{\begin{array}{cc} +1 & \text{if }{\hat{y}}_{i}\geq t\\ -1 & \text{if }{\hat{y}}_{i}<t \end{array}$. The threshold, *t*, was varied over the entire range of ${\hat{y}}_{i}$, i.e., from $\underset{i}{\text{min}}\left({\hat{y}}_{i}\right)$ to $\underset{i}{\text{max}}\left({\hat{y}}_{i}\right)$) and the ROC (receiver operating characteristics) curve was created by plotting true positive rate (TPR) against false positive rate (FPR) of classification at various settings of threshold *t*. The area under the ROC curve was computed by numerical integration using trapezoidal approximation.

$$\begin{array}{cccc} & & \text{Predicted} & \text{Class}\\ & & +1 & -1\\ \text{Actual} & +1 & TP & FN\\ \text{Class} & -1 & FP & TN \end{array}$$

$$TPR=\frac{TP}{TP+FN},$$

$$FPR=\frac{FP}{FP+TN}$$

### Matthews correlation coefficient

For binary classification using support vector classification (SVC), we computed the classification accuracy in terms of Matthews correlation coefficient (MCC) defined as: $MCC=\frac{TP\times TN-FP\times FN}{\sqrt{\left(TP+FP\right)\left(TP+FN\right)\left(TN+FP\right)\left(TN+FN\right)}}$. MCC is generally regarded as a balanced measure taking into account the classification accuracy in both positive and negative classes.

### Hierarchical network analysis

Our method of constructing a hierarchy from a directed regulatory network is inspired by existing methods in the literature but is more suitable for identifying few important regulators in the network. Bhardwaj, et al., [19] reported a simple method of building a hierarchy in gene networks where they organized TFs into three different layers—TFs which are not regulated by other TFs were placed in the top layer, TFs with no out-going edges to other TFs were in the lowest layer, and TFs which both regulate other TFs and are themselves regulated by other TFs were in the middle layer. This method was extended to include miRNAs by Cheng, et al., [12] where miRNAs were placed in separate layers above the TFs they regulate and in-between the layers of TFs. We found that in our network there were no TFs which could be classified in the top layer. A simple hierarchy was in-fact impossible to construct due to the presence of loops (TFs regulating each other) where it is hard to distinguish which regulator should be on the top or below.

We identified the hierarchy of genes based not only on their immediate regulatory interactions but also on their overall importance within the network. We used K-shell (also known as K-core) decomposition, a classical method in graph theory [69] which has been used to obtain a hierarchy of nodes based on their degree characteristics in bacteria and yeast GRNs [42,43]. Each K-shell was obtained by successively removing nodes of degree K beginning with degree 1. This was repeated until a final irreducible K-core was left. Recent literature has found that the K-core contains the nodes that have the greatest potential for information spread within the network [45]. Thus using K-core we retained the most essential central nodes for consideration.

## Supporting Information

S1 Text Additional analyses. Provides additional analyses on (1) Four different MCF-7 E2 vs. Control public datasets used to build the MCF-7 ER network, (2) Literature support for regulatory nodes in the MCF-7 ER network, (3) Comparison of MCF-7 ER network with random networks.
(DOCX)

S1 Data Datasets and source code. Contains integrated human TF-miRNA-mRNA network dataset, MCF-7 ER network dataset, source code for LR, PCA, SVR and SVC analysis on MCF-7 ER network.
(ZIP)
