# Supplementary material for: Automated Identification of Core Regulatory Genes in Human Gene Regulatory Networks
Source: PLoS Comput Biol. 2015 Sep 22;11(9):e1004504. doi: 10.1371/journal.pcbi.1004504 (PMC4578944; doi:10.1371/journal.pcbi.1004504)
Supplement: S1 Text — Provides additional analyses on (1) Four different MCF-7 E2 vs. Control public datasets used to build the MCF-7 ER network, (2) Literature support for regulatory nodes in the MCF-7 ER network, (3) Comparison of MCF-7 ER network with random networks. (DOCX) [file pcbi.1004504.s001.docx]

# This file contains additional figures and tables to supplement the article Narang V et al., “Automated Identification of Core Regulatory Genes in Human Gene Networks”, 2015

**(1) Four different MCF-7 E2 vs. Control public datasets used to build the MCF-7 ER network**

| 12hrs | 24hrs | No. of genes |
| --- | --- | --- |
| Up | Up | 806 |
| Up | Down | 0 |
| Down | Up | 0 |
| Down | Down | 567 |


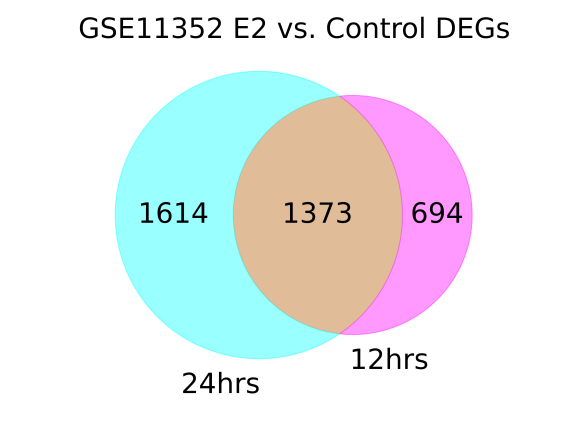


(a) (b)

Supplementary Figure 1: (a) Overlap between lists of differentially expressed genes (FDR < 0.05) at 12hr and 24hr time points in GSE11352 dataset, (b) Directions of fold changes of 1373 common genes at 12hrs and 24hrs.


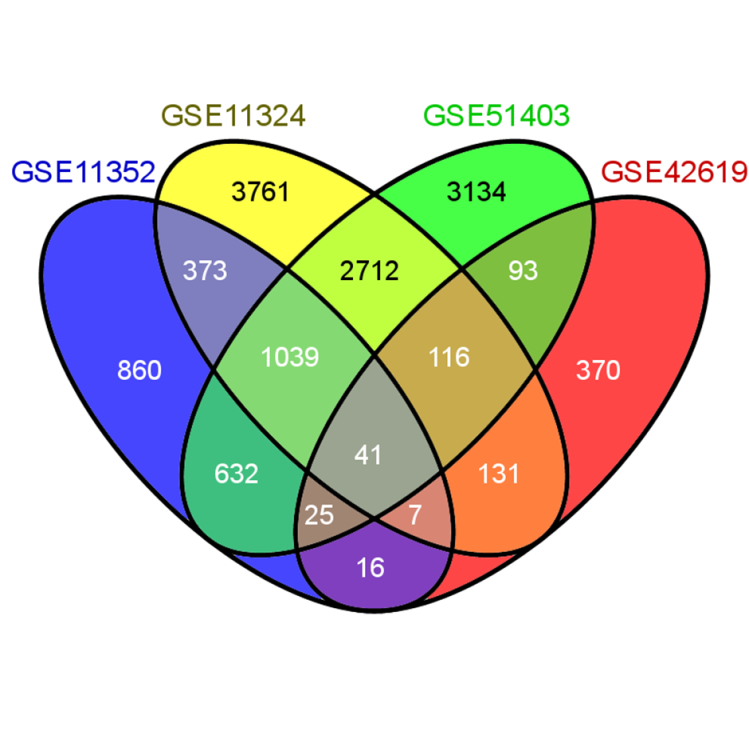


Supplementary Figure 2. Overlap of differentially expressed genes with FDR < 0.05 in various MCF-7 E2 vs. Control datasets.

**(2) Literature support for regulatory nodes in the MCF-7 ER network**

Supplementary Table 1. Google Scholar was used to perform a query using the format ‘+“MCF-7” +estrogen +regulator’ where regulator is the gene symbol. The number of results (publications) returned by Google Scholar is shown here as a quantitative measure of the relevance of a regulator to the MCF-7 ER network. For some regulators whose official gene symbol is not as popular as an alternative name, the alternative name was used to perform the query.

| **Molecule** | **Rank** | **Original Search** | **Alternate Name** | **Alternate Search** | **Final Count** |
| --- | --- | --- | --- | --- | --- |
| ESR1 | 1 | 2270 | Estrogen Receptor | 84700 | 84700 |
| TP53 | 2 | 3670 | p53 | 26600 | 26600 |
| JUN | 3 | 21300 |  |  | 21300 |
| PGR | 4 | 3870 | Progesterone receptor | 19900 | 19900 |
| MYC | 5 | 16100 |  |  | 16100 |
| AR | 6 | NA | Androgen Receptor | 11000 | 11000 |
| FOS | 7 | 9180 |  |  | 9180 |
| BRCA1 | 8 | 7780 |  |  | 7780 |
| STAT3 | 9 | 4750 | STAT | 6460 | 6460 |
| STAT2 | 10 | 240 | STAT | 6460 | 6460 |
| E2F1 | 11 | 2410 | E2F | 4260 | 4260 |
| E2F6 | 12 | 110 | E2F | 4260 | 4260 |
| E2F3 | 13 | 328 | E2F | 4260 | 4260 |
| SMAD3 | 14 | 1320 | SMAD | 2690 | 2690 |
| FOXA1 | 15 | 986 | Forkhead | 2690 | 2690 |
| ARNT | 16 | 1900 |  |  | 1900 |
| GATA3 | 17 | 944 | GATA | 1820 | 1820 |
| GATA2 | 18 | 130 | GATA | 1820 | 1820 |
| MYB | 19 | 1590 |  |  | 1590 |
| CXCL12 | 20 | 1330 |  |  | 1330 |
| SP3 | 21 | 1260 |  |  | 1260 |
| ELF1 | 22 | 44 | Elongation Factor | 1190 | 1190 |
| TFAP2C | 23 | 126 | AP-2 | 1140 | 1140 |
| HDAC2 | 24 | 1080 |  |  | 1080 |
| TP63 | 25 | 82 | p63 | 1070 | 1070 |
| EZH2 | 26 | 991 |  |  | 991 |
| CDKN1A | 27 | 942 |  |  | 942 |
| JUNB | 28 | 746 | AP-1 | 9470 | 746 |
| HIF1A | 29 | 298 | HIF1 | 684 | 684 |
| REL | 30 | 680 |  |  | 680 |
| SRF | 31 | 574 | Serum response | 1030 | 574 |
| SMAD7 | 32 | 573 |  |  | 573 |
| EGR1 | 33 | 571 |  |  | 571 |
| PARP1 | 34 | 560 |  |  | 560 |
| ATF2 | 35 | 544 |  |  | 544 |
| SOX3 | 36 | 55 | SOX | 522 | 522 |
| NCOA3 | 37 | 511 |  |  | 511 |
| USF1 | 38 | 135 | USF | 432 | 432 |
| USF2 | 39 | 94 | USF | 432 | 432 |
| ATF3 | 40 | 418 |  |  | 418 |
| SOX9 | 41 | 399 |  |  | 399 |
| HMGB1 | 42 | 382 |  |  | 382 |
| ATF4 | 43 | 373 |  |  | 373 |
| ETS2 | 44 | 360 |  |  | 360 |
| RXRA | 45 | 331 |  |  | 331 |
| NME2 | 46 | 295 |  |  | 295 |
| HES1 | 47 | 295 |  |  | 295 |
| YBX1 | 48 | 40 | YB-1 | 292 | 292 |
| E2F2 | 49 | 279 |  |  | 279 |
| PPARA | 50 | 278 |  |  | 278 |
| RUNX3 | 51 | 241 |  |  | 241 |
| GATA4 | 52 | 232 |  |  | 232 |
| MEF2C | 53 | 183 |  |  | 183 |
| CREB1 | 54 | 180 |  |  | 180 |
| IRF1 | 55 | 172 |  |  | 172 |
| FOXC2 | 56 | 169 |  |  | 169 |
| EGR3 | 57 | 157 |  |  | 157 |
| TCF7L2 | 58 | 136 |  |  | 136 |
| MAFF | 59 | 132 |  |  | 132 |
| TAF1 | 60 | 126 |  |  | 126 |
| HBP1 | 61 | 123 |  |  | 123 |
| CEBPA | 62 | 120 |  |  | 120 |
| E4F1 | 63 | 114 |  |  | 114 |
| RAD21 | 64 | 104 |  |  | 104 |
| HSF2 | 65 | 95 |  |  | 95 |
| GABPA | 66 | 93 |  |  | 93 |
| RXRB | 67 | 84 |  |  | 84 |
| SREBF1 | 68 | 82 |  |  | 82 |
| MAFG | 69 | 82 |  |  | 82 |
| NR1H3 | 70 | 81 |  |  | 81 |
| ESRRA | 71 | 81 |  |  | 81 |
| MAFK | 72 | 75 |  |  | 75 |
| LMO2 | 73 | 72 |  |  | 72 |
| HEY2 | 74 | 69 |  |  | 69 |
| HSA-MIR-489 | 75 | 62 |  |  | 62 |
| MYBL1 | 76 | 62 |  |  | 62 |
| KLF10 | 77 | 59 |  |  | 59 |
| ILF3 | 78 | 56 |  |  | 56 |
| NFAT5 | 79 | 56 |  |  | 56 |
| SMC3 | 80 | 56 |  |  | 56 |
| ASCL1 | 81 | 46 |  |  | 46 |
| IRF2 | 82 | 44 |  |  | 44 |
| ELK4 | 83 | 43 |  |  | 43 |
| SIM1 | 84 | 42 |  |  | 42 |
| NFYA | 85 | 42 |  |  | 42 |
| MEIS2 | 86 | 38 |  |  | 38 |
| BCLAF1 | 87 | 37 |  |  | 37 |
| KLF11 | 88 | 36 |  |  | 36 |
| RFX5 | 89 | 31 |  |  | 31 |
| ZBTB4 | 90 | 30 |  |  | 30 |
| GRHL3 | 91 | 28 |  |  | 28 |
| SMARCC1 | 92 | 26 |  |  | 26 |
| CHD2 | 93 | 25 |  |  | 25 |
| PBX3 | 94 | 23 |  |  | 23 |
| HNRNPD | 95 | 20 |  |  | 20 |
| BHLHE40 | 96 | 20 |  |  | 20 |
| BATF | 97 | 20 |  |  | 20 |
| ZNF143 | 98 | 18 |  |  | 18 |
| THAP1 | 99 | 17 |  |  | 17 |
| TCERG1 | 100 | 16 |  |  | 16 |
| AFF4 | 101 | 15 |  |  | 15 |
| HSA-MIR-17-5P | 102 | 14 |  |  | 14 |
| FOXE3 | 103 | 12 |  |  | 12 |
| ZBTB33 | 104 | 11 |  |  | 11 |
| GABPB1 | 105 | 10 |  |  | 10 |
| MXD4 | 106 | 10 |  |  | 10 |
| PURB | 107 | 9 |  |  | 9 |
| FOXN2 | 108 | 8 |  |  | 8 |
| HSA-MIR-548M | 109 | 8 |  |  | 8 |
| HSA-MIR-1231 | 110 | 7 |  |  | 7 |
| CREB3L1 | 111 | 6 |  |  | 6 |
| HSA-MIR-653 | 112 | 6 |  |  | 6 |
| ZNF367 | 113 | 6 |  |  | 6 |
| GTF2F1 | 114 | 6 |  |  | 6 |
| CEBPZ | 115 | 5 |  |  | 5 |
| HSA-MIR-20A-5P | 116 | 5 |  |  | 5 |
| HSA-MIR-93-5P | 117 | 5 |  |  | 5 |
| HSA-MIR-942 | 118 | 5 |  |  | 5 |
| HSA-MIR-22-3P | 119 | 5 |  |  | 5 |
| HSA-MIR-941 | 120 | 4 |  |  | 4 |
| HSA-MIR-636 | 121 | 4 |  |  | 4 |
| HSA-MIR-25-3P | 122 | 4 |  |  | 4 |
| HSA-MIR-92A-3P | 123 | 4 |  |  | 4 |
| HSA-MIR-623 | 124 | 3 |  |  | 3 |
| NAA15 | 125 | 2 |  |  | 2 |
| HSA-MIR-548F | 126 | 2 |  |  | 2 |
| HSA-MIR-4800-5P | 127 | 0 | mir-4800 | 2 | 2 |
| WRNIP1 | 128 | 1 |  |  | 1 |
| HSA-MIR-106B-5P | 129 | 1 |  |  | 1 |
| TMEM229A | 130 | 0 |  |  | 0 |
| HSA-MIR-3190-5P | 131 | 0 |  |  | 0 |
| HSA-MIR-3652 | 132 | 0 |  |  | 0 |
| HSA-MIR-1292-5P | 133 | 0 |  |  | 0 |
| HSA-MIR-3917 | 134 | 0 |  |  | 0 |
| hsa-miR-4640-3P | 135 | 0 | mir-4640 | 0 | 0 |

**(3) Effect of modifying TF-target definition**

Supplementary Figure 3. A gene is defined as target of a TF if a TF binding site lies close to the gene’s transcription start site (TSS). The interval around the TSS to consider a valid TF-gene interaction is varied from ±250bp to ±4kb. Consequently the variation in the (a) total number of TF-gene interactions (or network edges), (b) TF out-degree distribution, and (c) mRNA in-degree distribution is shown.
